# Occupational correlates of smoking among urban transit operators: A prospective study

**DOI:** 10.1186/1747-597X-2-36

**Published:** 2007-12-20

**Authors:** Carol B Cunradi, Rob Lipton, Aniruddha Banerjee

**Affiliations:** 1 Prevention Research Center, Pacific Institute for Research and Evaluation, 1995 University Avenue, Suite 450, Berkeley, CA 94704, USA

## Abstract

**Background:**

Workers in blue-collar and service occupations smoke at higher rates than workers in white-collar and professional occupations. Occupational stress may explain some of the occupational class differences in smoking and quitting behavior. The purpose of this study is to investigate the contribution of occupational factors to smoking behavior over a ten year period among a multiethnic cohort of urban transit operators, while accounting for demographic factors and alcohol.

**Methods:**

The sample consists of 654 San Francisco Municipal Railway (MUNI) transit operators who participated in two occupational health studies and biennial medical examinations during 1983–85 and 1993–95. Workers who had initiated, increased, or maintained their smoking over the ten year period were compared to workers who remained non-smokers. Occupational factors included self-rated frequency of job problems (e.g., difficulties with equipment, passengers, traffic), job burnout (i.e., the emotional exhaustion subscale of the Maslach Burnout Inventory), time needed to unwind after work, and years employed as a transit operator. A series of logistic regression models were developed to estimate the contribution of occupational factors to smoking behavior over time.

**Results:**

Approximately 35% of the workers increased, initiated, or maintained their smoking over the ten-year period. Frequency of job problems was significantly associated with likelihood of smoking increase, initiation, or maintenance (OR = 1.30; 95% CI 1.09, 1.55). Black operators were significantly more likely to have smoked over the ten-year period compared to operators in other racial/ethnic groups.

**Conclusion:**

Understanding the role of work-related stress vis-à-vis smoking behavior is of critical importance for crafting workplace smoking prevention and cessation interventions that are applicable to blue-collar work settings, and for developing policies that mitigate occupational stress.

## Background

Smoking is the leading cause of preventable death in the U.S. [1]. Although rates of cigarette smoking have declined over the past decades, the rates of decline have varied by gender, ethnicity, and occupational status [2], resulting in significant tobacco-related health disparities [3]. Workers in blue-collar and service occupations, for example, continue to smoke at higher rates than workers in white-collar and professional occupations [4-6]. Evidence for the occupational gap in smoking rates is illustrated by a comparison of data from the 1978–80 and 1997 National Health Interview Surveys (NHIS) [2]. In the NHIS, white-collar workers included professional and technical occupations, managers and administrators, sales workers, and clerical workers. Blue-collar workers included craftsmen, operatives except transportation, transportation operatives and laborers. Service workers included public servants and private household workers. In 1978–80, blue-collar workers were 38% more likely to smoke cigarettes than were white-collar workers; by 1997, they were 75% more likely to do so. Likewise, in 1978–80, service workers were 17% more likely to smoke cigarettes than white-collar workers, but by 1997, they were 55% more likely to smoke [2]. In addition, data from the 1997 NHIS indicate that among smokers, a greater proportion of blue-collar workers (27.5%) than white-collar workers (18.0%) could be classified as heavy smokers (defined as smoking ≥ 25 cigarettes per day). Workers in blue-collar and service occupations are also less likely to quit smoking compared to white-collar workers. Among ever smokers in the 1997 NHIS, 51.3% of white-collar workers, 36.8% of blue-collar workers, and 32.8% of workers in service occupations were former smokers [2].

Occupational stress may be a key factor in explaining occupational class differences in smoking and quitting behavior. Past studies suggest that higher levels of job strain (i.e., high job demands and low job control) and perceived job stress are associated with increased intensity of smoking and decreased quitting [7-10]. Workers in blue-collar and service occupations may be more likely to experience occupational stress, and stress levels may make quitting more difficult [11,12]. The occupation of transit operator, relative to many other occupations, is very stressful, as documented through neuroendocrine elevations on the job vis-à-vis resting states [13]. Compared to other occupations, rates of smoking among transit operators have been found to be elevated. For example, respondents who were classified as motor vehicle operators in the third National Health and Nutrition Examination Survey (NHANES III), conducted from 1988–1994, had an estimated smoking prevalence of 41.5% [6]. In contrast, those classified as machine operators had an estimated smoking prevalence of 34.2%; freight, stock, and material movers had an estimated smoking prevalence of 25.2%, and the smoking prevalence of teachers was estimated at 12.2% [6].

The purpose of the current study is to investigate the contribution of occupational factors to smoking behavior over a ten year period among a multiethnic cohort of urban transit operators, while accounting for potentially confounding effects of demographic factors and alcohol. For example, previous analyses indicate that drinking is associated with occupational stress factors among transit operators [14-17]. In terms of the psychosocial workplace environment, understanding the role of work-related stress vis-à-vis smoking maintenance and cessation is of critical importance for crafting workplace smoking prevention and cessation interventions that are applicable to blue-collar work settings [18]. The analysis is based on data collected from transit operators at the San Francisco Municipal Railway (MUNI), the transit system for the City of San Francisco, who participated in two cross-sectional occupational health and safety surveys in 1983–85 and 1993–95 that were administered in conjunction with a mandatory biennial medical exam. As the seventh largest public transit system in the U.S., MUNI is an important part of San Francisco's civic landscape, with an average weekday ridership of over 700,000 [19].

## Methods

### Sample and data collection

The sample for this study is based on San Francisco MUNI transit operators who participated in two different cross-sectional occupational health studies that occurred over a ten-year period. The details of the data collection process, and the algorithm for inclusion in the study sample, are described elsewhere [14,20], and are summarized below. The study's human subjects protocol was approved by the Institutional Review Board of the Pacific Institute for Research and Evaluation.

The first step in the data collection was part of a state-mandated commercial driver's license renewal examination at the Center for Municipal Occupational Safety & Health (CMOSH) clinic. All operators who underwent their mandatory biennial exam between December 1983 and September 1985 were eligible to participate. A total of 1,871 operators completed a self-reported health questionnaire, which was reviewed with a medical examiner, and each driver then received a complete physical examination. The second part of the data collection was conducted after the operator underwent re-licensing medical examination. At the request of the research staff, 1,450 operators agreed to complete a confidential, self-administered occupational and psychosocial questionnaire (77.5% response rate).

As with the 1983–85 survey, data for the 1993–95 survey data collection was integrated with the mandatory biennial re-licensing examination at the CMOSH clinic. All transit operators who underwent their examination between August 30, 1993 and September 29, 1995 were eligible to be included in the study (n = 1,974). This group includes virtually the entire population of MUNI transit operators. The operators completed a medical history form, which was reviewed with a medical examiner, and each driver then received a complete physical examination, for which a medical examination report was completed by the examining physician. The second part of the data collection was conducted after the operator underwent re-licensing medical examination. At the request of the research staff, 1,553 operators (78.7% response rate) completed a confidential, self-administered occupational and psychosocial questionnaire.

A total of 1016 workers participated in both occupational medical exams. Of these, 230 workers initiated, increased, or maintained their smoking between the 1983/85 and 1993/95 studies, and 424 workers remained non-smokers during the same time period. The current study analysis is based on these 654 workers. We had originally sought to model factors associated with smoking decrease among 82 workers who reported smoking during the 1983/85 study, and had either decreased their smoking level or quit by the 1993/95 study. The results, however, produced unstable estimates, probably due to small cell size on certain variables. In addition, 280 cases were excluded due to large amounts of missing survey data. In a comparison of workers with complete and missing data, no differences were observed for average age, years of being a MUNI operator, or percentage male. A greater proportion of workers with missing data, however, had initiated, increased or maintained their smoking between the 1983/85 and 1993/95 studies compared to workers with compete data (43% vs. 35%).

### Measures

Information on tobacco use and years of driving was collected as part of the 1983–85 and 1993–95 occupational medical exams. The variables on occupational factors and alcohol were obtained from the 1993–95 occupational health survey.

### Smoking behavior over 10 years (dependent variable)

As part of the medical exam in 1983–85 and 1993–95, operators were asked about current and past smoking. Current smokers were asked, "How many cigarettes do you smoke per day?" (< 1/2 pack; 1/2 pack; 1 pack; 1 1/2 packs; 2 packs or more). Former smokers were asked, "How many cigarettes did you USED to smoke per day?" (< 1/2 pack; 1/2 pack; 1 pack; 1 1/2 pack; 2 packs or more). Based on responses to these questions, operators were classified as either having initiated, increased, or maintained their smoking between 1983–85 and 1993–95, or as non-smokers during the same time period.

### Background characteristics

The age, gender and race/ethnicity of each respondent were collected. For the descriptive statistics, respondents were categorized as Asian/Filipino; Black; Hispanic; Caucasian; and Other. Since Black operators constitute half the sample, and due to small numbers of operators in some of the race/ethnicity categories, Black operators were compared to all non-Black operators in the multivariate analysis. It should be noted that as municipal transit operators employed by the same urban transit agency, the cohort is relatively homogenous regarding education and income level [15,21]. For example, the average worker completed one year post-high school education. In addition, the hourly wage for all transit operators is the same as governed by the union contract. Due to the low variability in education and income level, we therefore did not include these variables in the models.

### Alcohol consumption

As part of the self-administered questionnaire given after the re-licensing examination was completed, operators were asked, "In an average week, how much of the following do you drink?" They were asked to separately list the number of glasses of wine per week, number of cans or bottles of beer per week, and the number of drinks of liquor (e.g., gin, rum, vodka, whiskey) per week. Based on responses to these questions, an overall measure of alcohol quantity-frequency was calculated from which a measure of daily mean ounces of alcohol was derived.

### Occupational factors

Information on occupational factors was measured as follows:

#### (1) Years driving

The duration of professional driving as a transit operator was measured in years of service.

#### (2) Job problems

Survey participants were asked to rate the past-year frequency of the following potential job problems: Equipment problems, problems with fares and transfers, too many passengers, problems caused by passengers, problems caused by coworkers, problems with supervisor, long or odd hours, written up for rule violation, unfairly written up for rule violation, minor accident with no injuries, serious accident with injuries, accident that is your fault, serious traffic or road problems, problems with other vehicles, crimes against you while on duty, crimes against your passengers, problems communicating with central control, poor access to restrooms, not maintaining run schedule. Response categories ranged from "daily" = 5 to "never" = 1. Internal reliability was Cronbach's α = .86. A summary index for frequency of job problems was calculated by adding across events and dividing by the number of items. This list of job problems was developed by conducting individual interviews and focus groups among MUNI transit operators [22], and previous studies have found a significant relationship between this measure and alcohol-related behavior [21,14,20]

#### (3) Time to unwind after work

Survey participants were asked, "How much time does it actually take to unwind and relax after work?" Response categories were: less than an hour = 1; about an hour = 2; several hours = 3; I can rarely unwind or relax = 4. In previous cross-sectional analysis, length of time to unwind was significantly associated with level of alcohol consumption in the after-work period [17].

#### (4) Burnout

Survey participants completed the emotional exhaustion subscale of the Maslach Burnout Inventory (MBI) [23], which consists of the following nine statements: I feel emotionally drained from my work; I feel used up at the end of the workday; I feel fatigued when I get up in the morning and have to face another day on the job; Working with people all day is really a strain for me; I feel burned out from my work; I feel frustrated by my job; I feel I'm working too hard on my job; Working with people directly puts too much stress on me; I feel like I'm at the end of my rope. Response categories ranged from "every day" = 7 to "never" = 1. Internal reliability was Cronbach's α = .94. Burnout scores are computed by summing across items and dividing by number of items. Higher scores reflect greater levels of burnout (i.e., emotional exhaustion). In cross-sectional analysis, burnout was associated with elevated risk of alcohol dependence among transit operators [15].

### Analytic strategy

As an initial step, descriptive statistics (percentages, means and standard deviations) were calculated to compare the characteristics of operators who increased, initiated, or maintained smoking to operators who remained non-smokers. Mantel-Haenszel chi-squares were calculated for categorical variables, and ANOVAs were run to compare means of continuous variables. For the multivariate analysis, a series of logistic regression models were developed to estimate the contribution of occupational factors to smoking behavior over 10 years. For each variable, the beta coefficient, odds ratio and 95% confidence interval was calculated, along with a corresponding p-value. In the first model, demographic factors (gender, race/ethnicity, and age) were included. Since drinking and smoking often co-occur, daily mean ounces of alcohol were entered into the second model. For the third and final model, occupational factors (burnout, frequency of job problems, time needed to unwind after work, and years driving), demographic factors, and alcohol were included. All analyses were conducted with the S Plus statistical software program.

## Results

### Sample characteristics by smoking status (Table 1)

**Table 1:** Characteristics of Study Subjects (N = 654) by Smoking Status

**Category**		**Increase, Initiate, or Maintain Smoking (n = 230)**		**Non-smoker (n = 424)**		**Mantel-Haenszel Chi-Square**	
		**No.**	**%**	**No.**	**%**	**Value**	**Probability**

**Gender**	Male	204	(88.7%)	396	(93.4%)	5.67	0.02
	Female	26	(11.3%)	28	(6.6%)	5.32	0.03
**Race/ethnicity**	Asian/Filipino	30	(13.0%)	86	(20.3%)	7.32	0.04
	Black	132	(57.4%)	200	(47.2%)	6.50	0.02
	Hispanic	22	(9.6%)	50	(11.8%)	3.32	0.09
	White	41	(17.8%)	77	(18.2%)	2.89	0.10
	Other	5	(2.2%)	11	(2.6%)	1.56	0.24

		**Mean**	**Standard Deviation**	**Mean**	**Standard Deviation**	**ANOVA**	
						**F value**	**Prob. > F**

**Age (years)**		49.80	(5.48)	50.61	(5.94)	2.66	0.10
**Alcohol**		0.42	(0.76)	0.30	(0.62)	4.53	0.03
**Unwind time**		1.80	(0.82)	1.68	(0.80)	3.22	0.07
**Job problems**		1.69	(1.14)	1.81	(1.17)	1.57	0.21
**Burnout**		1.77	(1.52)	1.62	(1.39)	1.71	0.19
**Years driving**		18.12	(5.71)	18.47	(6.30)	0.69	0.40

Approximately 35% (230/654) of the operators in the study increased, initiated, or maintained their smoking over the ten-year period. Of these 230 workers, 24 workers (10.4%) increased their smoking, 40 workers (17.4%) initiated smoking, and 166 workers (72.2%) maintained their smoking level. Among female operators, a greater proportion increased, initiated or maintained smoking than remained non-smokers (11.3% vs. 6.6%; Mantel-Haenszel Chi-square 5.32, degrees of freedom [df] = 1; *p*-value < 0.05). Regarding race/ethnicity, a greater proportion of Black operators increased, initiated or maintained smoking compared to those who remained non-smokers (57.4% vs. 47.2%; Mantel-Haenszel Chi-square 6.50, df = 1, *p*-value < 0.05). There was little difference in age, years driving, time needed to unwind, burnout scores or job problems between the smoking and non-smoking operators. Smokers drank more alcohol on average than non-smokers (F-value = 4.53, *p*-value < 0.05).

### Multivariate models of smoking increase, initiation & maintenance (Table 2)

**Table 2:** Logistic Regression Models for Smoking Increase, Initiation & Maintenance (N = 654)

		**Model 1**				**Model 2**				**Model 3**			
**n = 654**	**DF**	**B**	**p-value**	**OR**	**95% CI**	**B**	**p-value**	**OR**	**95% CI**	**B**	**p-value**	**OR**	**95% CI**
			
(Intercept)		0.987	0.196	2.68		0.863	0.261	2.37		0.901	0.293	2.46	
Male	1	-0.449	0.123	0.64	0.36, 1.13	-0.498	0.089	0.61	0.34, 1.08	-0.508	0.096	0.60	0.33, 1.09
Black	1	0.416	0.014	1.52	1.09, 2.11	0.413	0.015	1.51	1.08, 2.11	0.439	0.011	1.55	1.10, 2.18
Age (yr)	1	-0.028	0.056	0.97	0.94, 1.00	-0.026	0.073	0.97	0.95, 1.00	-0.029	0.086	0.97	0.94, 1.00
Alcohol	1					0.251	0.037	1.29	1.01, 1.63	0.235	0.054	1.26	1.00, 1.61
Burnout	1									0.114	0.116	1.12	0.97, 1.29
Job problems	1									0.263	0.004	1.30	1.09, 1.55
Unwind time	1									0.192	0.100	1.21	0.96, 1.52
Years driving	1									0.001	0.955	1.00	0.97, 1.03

DF = Degrees of freedom

OR = Odds Ratio

CI = Confidence Interval

The logistic regression results for Model 1 indicate that male operators were not more likely to increase, initiate or maintain smoking than female operators (Odds Ratio [OR] = 0.64; 95% Confidence Interval [CI] 0.36, 1.13, Wald chi-square 2.38, df = 1, *p*-value = 0.12). Black operators, however, were significantly more likely to remain smokers over the ten-year period than non-Black operators (OR = 1.52; 95% CI 1.09, 2.11, Wald chi-square 10.78, df = 1, *p *= 0.014). Operator age was not significantly associated with increased or decreased likelihood of increasing, initiating or maintaining smoking (OR = 0.97; 95% CI = 0.94, 1.00; Wald chi-square 5.04, df = 1, *p*-value = 0.056).

With the addition of mean daily ounces of alcohol to Model 2, the results seen in Model 1 did not change substantially. Compared to non-Black operators, Black operators remained significantly more likely to have increased, initiated or maintained smoking over the ten-year period (OR = 1.51; 95% CI 1.08, 2.11; Wald chi-square 10.05, df = 1, *p*-value = 0.015). Mean ounces of alcohol was significantly associated with increased risk for smoking increase, initiation, or maintenance (OR = 1.29; 95% CI 1.01, 1.63; Wald chi-square 3.65, df = 1, *p*-value = 0.037).

The logistic regression results for Model 3 show that after accounting for alcohol and occupational factors, Black operators remained significantly more likely to have smoked over the ten-year period compared to non-Black operators (OR = 1.55; 95% CI 1.10, 2.18; Wald chi-square 6.42, df = 1, *p*-value = 0.011). Mean daily ounces of alcohol was no longer statistically significant at the 0.05 level (OR = 1.26; 95% CI 1.00, 1.61; Wald chi-square 3.70, df = 1, *p*-value = 0.054). Regarding the occupational variables, burnout (OR = 1.12; 95% CI 0.97, 1.29; Wald chi-square 2.46, df = 1, *p*-value = 0.116), time to unwind (OR = 1.21; 95% CI 0.96, 1.52; Wald chi-square 2.71, df = 1, *p*-value = 0.10), and years driving (OR = 1.00; 95% CI 0.97, 1.03; Wald chi-square 0.01, df = 1, *p*-value = 0.955) were not significantly associated with likelihood of smoking over time. Frequency of job problems, however, was significantly associated with likelihood of smoking increase, initiation, or maintenance (OR = 1.30; 95% CI 1.09, 1.55; Wald chi-square 8.37, df = 1, *p*-value = 0.004).

## Discussion

Among a cohort of MUNI transit operators followed over ten year, the results indicate that approximately 35% were current smokers in 1993–95. When compared against smoking prevalence rates among California adults of comparable ages, the 1993–95 prevalence of smoking among MUNI transit operators is considerably higher. For example, the smoking prevalence in 1994 among California adults in the 25-44 and 45-64 year old age groups was approximately 18% [24]. These age groups encompass the ages of MUNI operators. Similarly, the 1993–95 smoking prevalence of Black MUNI operators within the cohort was nearly double the smoking prevalence among adult Blacks in California during 1993 (40% vs. 21%) [24].

A critical research issue is to determine what factors underlie the occupational class differences in smoking maintenance and cessation. Novotny et al. [25], in an analysis of the 1985 National Health Interview Survey, found no significant occupational class differences in a multivariate analysis of ever smoking, former smoking, and heavy smoking. In contrast, Giovino et al. [2] found that occupational class significantly predicted current smoking among participants in the 1997 NHIS (n = 19,935) after controlling for age, gender, educational attainment, and race/ethnicity. They found that the adjusted odds ratio for current smoking was 1.3 for workers in blue-collar and service occupations (reference group white-collar). If occupational class is independently related to current smoking, then factors such as occupational stress can be important targets in efforts to reduce occupational class disparities in smoking [2]. The current findings suggest that occupational stress factors are associated with smoking behavior over ten years among a cohort of urban transit operators. Because the occupational work environment can be modified, these findings have important implications for smoking prevention and quitting [7]. Moreover, the study findings linking frequency of job problems and alcohol use with smoking are in accord with a recent review of 50 years of international occupational health research on bus drivers [26] that noted the evidence linking the occupational stressors of bus drivers with substance use, including alcohol, tobacco and drugs. The study finding regarding increased risk of smoking among Black transit operators compared to those in other racial/ethnic groups, however, may not be generalizable to other transit agencies in the U.S. or in other countries.

The results indicate that Black operators were significantly more likely to initiate, increase, or maintain smoking over the ten-year study period compared to non-Black operators, even after adjusting for gender, age, occupational factors and alcohol. This is in contrast to some of the findings from the 2000 National Health Interview Survey reported by Barbeau et al. [27]. Their findings showed that Non-Hispanic Blacks were significantly less likely to be current smokers compared to non-Hispanic whites. In addition, current cigarette smoking among adults age 18 and older was most likely among men, those between ages 25 and 44 years old, and those with lower levels of education and income. Consistent with the occupational stress hypothesis, those employed in service industry or blue-collar jobs were more likely to be current smokers compared to white collar workers.

A potential limitation of this study, given the decline in smoking prevalence over the past decade, is whether or not factors that predict smoking behavior from 1983–85 to 1993–95 are related to smoking behavior a decade later or beyond. From 1970 to 1990, smoking prevalence fell by 0.6 percentage points per year. From 1995–2002, smoking prevalence declined at a rate only slightly greater than half that of the earlier period [28]. Although the rates of decline have decreased, there is no theoretical or empirical evidence to suggest that the factors associated with smoking behavior among blue-collar workers have changed over the last 10 or 20 years. In fact, Mendez and Warner [28] state that, "the general dynamics that govern adult smoking prevalence exhibit a large degree of inertia and are likely to prevail for years to come."

Another potential limitation concerns bias associated with the self-report measurement of alcohol and tobacco. For example, despite assurances of confidentiality, drinkers (especially heavy drinkers) may have underreported their alcohol consumption. This would result in an underestimation of consumption, and an observed distribution of consumption that is somewhat flatter than the actual distribution. Assuming a true association between alcohol and the outcome of interest, this underestimation would most likely lead to an attenuation of the association. Likewise, no biomarkers (e.g., expired-air carbon monoxide, the nicotine metabolite cotinine and/or thiocyanate) were available to confirm self-reported tobacco use. Based on studies in which both self-reported tobacco use and biomarkers are collected, there is evidence that heavy smokers who report reducing their smoking do not show lower levels of biomarkers at follow-up [29]. This suggests compensatory smoking or under-reporting of the true amount of smoking may occur.

Finally, because data on the psychosocial work factors (burnout, job problems, time to unwind) were collected at the second time point (the 1993/95 survey), it is not possible to ascribe causality regarding smoking behavior to these factors. It is plausible, for example, that smoking behavior might result in poor relations with colleagues and members of the public, thus contributing to a poor psychosocial work environment. Similarly, it is possible that those with the worst work environments or those who developed smoking-related illnesses left the workforce during the 10-year follow-up period, thus biasing any finding towards the null. Reverse causation, social selection or healthy worker effects should therefore be considered when interpreting the findings of this study.

This study also has a number of strengths. First, the analysis is based on a sizeable, multiethnic occupational blue-collar cohort that could serve as an exemplar of occupational and environmental stress [30]. For example, urban transit operators are typically exposed to multiple stressors (e.g., pollution, traffic congestion, ergonomic problems, competing time demands) as highlighted by U.S. and international research (see [31,32]). Second, this study was able to follow smoking behavior of the cohort over a ten-year period. Although not originally designed as a longitudinal study, the participation of MUNI operators in two cross-sectional studies over ten years permitted prospective assessment of smoking among those workers for whom data were available at both time points. Third, the analysis tested measures of the psychosocial work environment, as well as mean alcohol consumption, in relation to smoking behavior over time. Since drinking and smoking often co-occur [33], it is important to account for the potentially confounding role of alcohol. Previous cross-sectional analyses of MUNI transit operators, for example, have found drinking to be associated with occupational stress factors [14,15].

## Conclusion

The results have important policy implications for smoking prevention and quitting. As noted by Ragland and colleagues [34], workplace interventions that address both individual and environmental factors are most likely to have a positive impact on the health outcomes of transit operators. Individual interventions could include smoking cessation and stress management programs. Involvement of Employee Assistance Program and Union or Peer Assistance Program personnel could be enlisted to help implement these worksite health promotion strategies. Numerous environmental policies have been proposed that could reduce factors that contribute to transit operator stress [34], such as dedicated transit areas, reduction of non-transit vehicles in downtown areas, transit flow strategies, and ergonomic evaluation and redesign. Multilevel interventions that go beyond focusing solely on individuals may be most effective in reducing smoking and problem drinking, increasing coping ability, and optimizing the health and well-being of transit operators.

## Competing interests

The author(s) declare that they have no competing interests.

## Authors' contributions

CC conceived of the study and drafted the manuscript. RL participated in its design and coordination and helped to draft the manuscript. AB participated in the design of the study and performed the statistical analysis. All authors read and approved the final manuscript.
